# Index Finger Pointing (Likely a Subtle Form of Hand Dystonia): Prevalence Across Movement Disorders

**DOI:** 10.3389/fneur.2018.00542

**Published:** 2018-07-04

**Authors:** Ana Vives-Rodriguez, Elan D. Louis

**Affiliations:** ^1^Division of Movement Disorders, Department of Neurology, Yale School of Medicine, Yale University, New Haven, CT, United States; ^2^Center for Neuroepidemiology and Clinical Neurological Research, Yale School of Medicine, Yale University, New Haven, CT, United States; ^3^Department of Chronic Disease Epidemiology, Yale School of Public Health, Yale University, New Haven, CT, United States

**Keywords:** movement disorders, neurological examination, dystonia, Parkinson's disease, essential tremor

## Abstract

**Objective:** To investigate the prevalence of index finger pointing (IFP) while walking, which is likely a subtle form of hand dystonia, in cranio-cervical focal dystonia syndromes, Parkinson's disease (PD), essential tremor (ET), and controls.

**Methods:** We recruited patients with an established diagnosis of PD, dystonia, or ET and healthy controls. All participants were videotaped while walking. Videotapes were evaluated by the authors, blinded to diagnosis, to assess the presence or absence of IFP.

**Results:** Two-hundred-fifty participants included 50 dystonia, 50 PD, 80 ET and 70 controls. IFP was present in 29/250 (11.6%) participants: 10 dystonia (20.0%), 8 PD (16.0%), 8 ET (10.0%), and 3 controls (3.8%) (*p* = 0.03). There was a significant evidence of a trend in the odds of having this sign among disorders with higher risk of dystonic features (dystonia>PD>ET>control; test for trend = 0.004). Among the 180 patients (dystonia, PD, and ET, i.e., excluding the 70 controls), IFP was present in 26 (14.4% prevalence).

**Conclusion:** IFP during gait, likely a subtle form of hand dystonia, was observed in 14.4% of movement disorder patients. The highest prevalence was in dystonia, the second highest in a disease that is often accompanied by dystonia (PD), a lower prevalence among individuals with a disease that is rarely accompanied by dystonia (ET), and the lowest in controls.

## Introduction

Movement disorders, including Parkinson disease (PD), essential tremor (ET) and dystonia, are common conditions encountered in neurological practices. Their clinical presentation is often variable and complex. To aid in diagnosis, the neurologist needs to rely on meticulous clinical evaluation.

During clinical practice, we observed that several patients with PD held one of their index fingers in a pointing position (i.e., pointing downwards) while walking. This is likely a subtle form of hand dystonia that has not been formally described and its prevalence is unknown among different movement disorders. Here we aimed to investigate the prevalence of index finger pointing (IFP) during gait in patients with PD, cranio-cervical focal dystonia, ET and healthy controls.

## Methods

Participants were recruited from September 2016 to October 2017 from two sources: (1) patients with an established diagnosis of ET, PD, or idiopathic dystonia followed in the Movement Disorders Clinic at Yale School of Medicine and seen consecutively by AV-R, or (2) ET participants enrolled consecutively during the same period in an NIH-funded study of ET. Healthy controls were recruited consecutively from: (1) the same Movement Disorders Clinic (spouses of the consecutive PD and dystonia patients) and (2) from an NIH-funded case-control study of ET during the same period. There were no refusals.

Initially, we hypothesized that the IFP sign would be a sign of basal ganglia dysfunction. Therefore, we decided to compare two different disease groupings: Group 1 (PD and dystonia, both of which are associated with basal ganglia dysfunction) vs. Group 2 (ET and healthy controls, neither of which are known to be associated with basal ganglia dysfunction). The initial goal was to enroll 100 per group (200 enrollees in total); however, pilot data on the first 50 enrollees indicated that 150 would be needed in the ET+control group (i.e., 250 enrollees in total).

The study was approved by the Yale University Ethics Review Board; all enrollees signed written informed consent. All patients provided written and signed informed consent to be videotaped.

Patients all had an established diagnosis of ET, PD, or idiopathic dystonia made by a movement disorders specialist. Only patients previously diagnosed with cranio-cervical focal dystonia (i.e., torticollis, blepharospasm, Meige syndrome) were included in the dystonia group. Furthermore, to avoid complicating our interpretation of IFP, we did not enroll patients with any upper limb dystonia (e.g., spooning of hand during arm extension) observed on neurological examination prior to the evaluation of IFP, described below. Exclusion criteria included history of exposure to dopamine blocking agents, surgery, or trauma involving the arms, or prior deep brain stimulation surgery. For healthy controls, exclusion criteria included a family history of ET, PD, or dystonia, a diagnosis of a movement disorder, or use (prior or current) of dopamine blocking agents.

Clinical and demographic information was collected, including age, gender, duration of illness, symptomatic treatment used, Hoehn and Yahr (H&Y) staging (1) and time of last dose of dopaminergic therapy (for PD patients). After their neurological examination, participants were videotaped while walking along a straight corridor (30 feet, forwards and back twice). Videotapes were independently evaluated by the two authors (EDL, a senior movement disorders neurologist and AV-R, a movement disorders fellow) to establish the presence or absence of IFP. IFP was defined as extension of the index finger and partial or full flexion of the other digits; it could be present intermittently during the assessment (Video 1). When there were disagreements, the videotape was co-reviewed and a final consensus was reached.

Analyses were performed in Stata (Version IC 15.1). A Fisher exact test was used to examine group differences in prevalence of IFP (significance *p* = 0.05). A logistic regression model (test for trend) evaluated the relationship between diagnosis (independent variable coded as dystonia = 4, PD = 3, ET = 2, control = 1) and presence or absence of IFP (dependent variable). We evaluated the association between presence of IFP and clinical variables using Student's *t*-tests, Wilcoxon rank-sum tests (when skewness was observed) and chi-square tests; these analyses were stratified by diagnosis.

## Results

Two-hundred-fifty individuals were enrolled: 50 PD patients, 50 dystonia patients, 80 ET patients, and 70 healthy controls (Table 1). Most patients with PD were H&Y stage 2. Most dystonia patients had isolated cervical dystonia but we included six patients with isolated cranial dystonia. Thirty-nine (78.0%) PD patients were taking levodopa. The ET group had the longest duration of symptoms (median = 39 years).

**Table 1:** Clinical characteristics of 250 participants.

**Characteristics**	**Parkinson's disease (*n* = 50)**	**Dystonia (*n* = 50)**	**Essential tremor (*n* = 80)**	**Controls (*n* = 70)**
Age in years	67.4 ±7.5	62.1 ±11.3	74.1 ±11.7	57.3 ±12.5
Female gender^*^	15 (30.0)	41 (82.0)	44 (55.0)	29 (41.4)
Duration of symptoms (years)	Median: 4 (IQR: 2–4)	Median: 16 (IQR: 5–21)	Median: 39 (IQR: 19–58)	Not applicable
Clinical features^*^	H&Y stage: 1: 4 (8.0) 2: 43 (86.0) 2.5: 3 (6.0)	Cervical dystonia: 44 (88.0) Blepharospasm: 3 (6.0) Meige syndrome: 3 (6.0)	Not applicable	Not applicable
Symptomatic treatment used^*^	Levodopa: 39 Dopamine Agonists: 12 Amantadine: 6 MAO inhibitor: 8 COMT inhibitor: 2 No treatment: 6	Botulinum toxin: 41 Baclofen: 2 Tizanidine: 1 Clonazepam: 1 No treatment: 9	Propranolol: 24 Primidone: 7 Benzodiazepines: 3 Gabapentin: 3 No treatment: 26 Combination: 17	Not applicable

* Values represent number (percentage).

*IQR, interquartile range; H&Y, Hoehn and Yahr stage; MAO, monoamine oxidase; COMT, catechol-O-methyl transferase*.

IFP was present in 29/250 (11.6%) participants: 10/50 dystonia patients (20.0%), 8/50 PD patients (16.0%), 8/80 ET patients (10.0%), and 3/70 healthy controls (3.8%) (Fisher exact test *p* = 0.03). The highest prevalence of IFP was in the group with idiopathic dystonia, the second highest in individuals with a disease that can be accompanied by dystonia (PD), a lower prevalence among individuals with a disease that is rarely and arguably accompanied by dystonia (ET), and the lowest prevalence among healthy controls (dystonia>PD>ET>controls) (test for trend = 0.004). IFP was not associated to a significant degree (*p* < 0.05) with demographic or clinical variables (Table 2).

**Table 2:** Association of clinical variables with presence of index finger pointing (IFP) in analyses stratified by diagnosis.

	**IFP present**	**IFP absent**	***p*-value**
**Dystonia**	*n* = 10	*n* = 40	
Age (years)	65.3 ± 9.2	61.3 ± 11.8	0.32
Female gender^*^	10 (100)	31 (77.5)	0.17
Duration of illness (years)	Median: 13 (IQR:5–21)	Median: 19 (IQR:16–20)	0.51
**Parkinson's disease**	*n* = 8	*n* = 42	
Age (years)	67.3 ± 5.9	67.4 ± 7.8	0.95
Female gender^*^	4 (50.0)	10 (23.8)	0.13
Duration of illness (years)	Median: 2.3 (IQR:1.25–3.5)	Median: 4.5 (IQR:3–8)	0.05
Hoehn and Yahr stage	Stage 1: 2 (4.8)	Stage 1: 2 (25.0)	0.09
	Stage 2: 38 (90.4)	Stage 2: 5 (62.5)	
	Stage: 2.5: 2 (4.8)	Stage: 2.5: 1 (12.5)	
Time of last medication dose (hours)	Median: 2 (IQR:1–2)	Median: 2.5 (IQR:1.5–3.5)	0.34
**Essential tremor**	*n* = 8	*n* = 72	
Age (years)	70.3 ± 7.0	74.5 ± 12.0	0.33
Female gender^*^	5 (62.5)	39 (54.2)	0.65
Duration of illness	41 (IQR:21.5–49)	36.5 (IQR:19–58)	0.88
**Healthy controls**	*n* = 3	*n* = 67	
Age (years)	67.0 ± 14.2	56.9 ± 12.4	0.17
Female gender^*^	2 (66.6)	27 (40.3)	0.36

* Values represent number (percentage).

*IQR, interquartile range*.

Eight PD patients had IFP. The sign was present on the more affected side in 2 (25.0%), the less affected side in 5 (62.5%) and equally on both sides in 1 (12.5%). Five (62.5%) of 8 PD patients with IFP were taking carbidopa-levodopa but only three of 8 (37.5%) were experiencing motor fluctuations; hence, the majority (5 of 8 PD patients, 62.5%) were neither on carbidopa-levodopa or experiencing motor fluctuations. Of the three experiencing motor fluctuations, 2 were “on” and one was “off” during our examination of IFP.

## Discussion

We documented a 14.4% prevalence of IFP among patients with PD, ET, and craniocervical dystonia (i.e., 26/180), although its prevalence was graded across these disorders. Three percent of healthy controls also presented with the sign. There was significant evidence of a trend in the odds of having this sign among disorders with higher risk of dystonic features (dystonia>PD>ET>controls).

In PD, dystonia has been reported, with focal dystonia involving the foot, arm-forearm, neck and trunk most frequently described (2–4). Dystonia is frequently described as a motor complication related to chronic dopaminergic therapy. In our cohort, 5 (62.5%) of 8 PD patients with IFP were taking carbidopa-levodopa but only three of 8 (37.5%) were experiencing motor fluctuations; hence, the majority (5 of 8 PD patients, 62.5%) were neither on carbidopa-levodopa or experiencing motor fluctuations. Only two (25.0%) were “on” during our examination of IFP. Likewise, in our cohort, there was no association between the presence of IFP and the time of the last dose of dopaminergic therapy. Similarly, an association between H&Y stage and the presence of IFP could not be established.

Even though IFP was present more frequently in dystonia and PD patients, 10% of our ET patients presented with the sign. This is not unexpected considering that 10.7% of patients with ET have been reported to have subtle features of dystonia (5). Indeed, in the most recent consensus statement of the International Parkinson and Movement Disorder Society, the term *ET plus* was adopted to describe ET patients with concomitant mild neurological signs, including mild dystonic posturing (6).

One question is whether in patients with undeclared diagnoses (difficult-to-diagnose tremors), this sign could be of differentiating value. For example, what is its value in the distinction between tremors of dystonic origin vs. ET? What is its prevalence in patients with isolated head tremor with no dystonic features? Could it be an early marker to help distinguish emerging PD from controls? The current study has brought attention to this sign; future studies should evaluate its utility in a myriad of different clinical settings.

Several limitations of the study need to be discussed. First, even though the videotape review was blinded to intake diagnosis, in some instances, features of PD or dystonia were noticeable. Second, we did not gather information on foot dystonia, given its low prevalence in ET and cranio-cervical dystonia. However, it would be interesting to examine the presence of other such subtle features of dystonia in cases with IFP. Third, given the different natural courses of these disorders, there can be considerable variability in disease duration in typical cohorts, as there was in ours (e.g., median: PD 4 years, dystonia 16 years and ET 39 years). This is also to be expected since patients with PD will generally seek medical attention at an earlier point compared to patients with dystonia and ET. However, the presence of IFP was not associated to a significant degree with duration of illness in any of the disorders enrolled in this cohort (Table 2); hence, differences in prevalence of IFP across these conditions was likely not to have been an artifact of differences in disease duration across these conditions. Fourth, even though there is an ordering in the prevalence of IFP among cases (dystonia>PD>ET>controls), we acknowledge that the differences were small in several instances. Finally, our findings are restricted to the most prevalent movement disorders; future studies should include other disorders characterized by basal ganglia dysfunction such as Huntington's disease, Wilson's disease and Parkinson-plus syndromes. A longitudinal follow-up of healthy controls would allow us to establish whether this could be an early clinical marker of disease.

In summary, the diagnosis of movement disorders can be challenging. Subtle neurological signs, when identified, may aid the neurologist who is performing a meticulous examination. To our knowledge, the prevalence of IFP, which is likely a form of hand dystonia during gait, has not been formally described or quantified across different movement disorders. In this cohort, we document an 14.4% prevalence among patients with PD, ET, and cranio-cervical dystonia. Indeed, the highest prevalence of IFP was in the group with dystonia, the second highest in individuals with a disease that is often accompanied by dystonia (PD), a lower prevalence among individuals with a disease that is rarely and arguably accompanied by dystonia (ET), and the lowest prevalence among healthy controls. The current study has brought attention to this sign; future studies should evaluate its utility in a myriad of different clinical settings.

## Author contributions

AV-R was in charge of drafting/revising the manuscript for content, including medical writing for content, study design, collection of data, analysis and interpretation of data. EL was in charge of drafting/revising the manuscript for content, including medical writing for content, study concept and design, analysis and interpretation of data.

### Conflict of interest statement

EL receives/has received research support from NIH/NINDS. The remaining author declares that the research was conducted in the absence of any commercial or financial relationships that could be construed as a potential conflict of interest.

## Supplementary material

Video 1 Examples of IFP while walking. The first patient has a diagnosis of cervical dystonia. IFP is present in the left hand. The second patient has a diagnosis of Parkinson's disease. IFP is present in the right hand.
